# Marine-Inspired Approaches as a Smart Tool to Face Osteochondral Regeneration

**DOI:** 10.3390/md21040212

**Published:** 2023-03-28

**Authors:** Anna Tampieri, Elizaveta Kon, Monica Sandri, Elisabetta Campodoni, Massimiliano Dapporto, Simone Sprio

**Affiliations:** 1 Institute of Science, Technology and Sustainability for Ceramics, ISSMC-CNR (Former ISTEC-CNR), 40129 Faenza, Italy; 2 Humanitas Research Hospital, 20089 Rozzano, Italy

**Keywords:** osteochondral scaffold, hybrid materials, marine-derived ingredients, fucoidans, mutable collagen, apatites, 3D printing

## Abstract

The degeneration of osteochondral tissue represents one of the major causes of disability in modern society and it is expected to fuel the demand for new solutions to repair and regenerate the damaged articular joints. In particular, osteoarthritis (OA) is the most common complication in articular diseases and a leading cause of chronic disability affecting a steady increasing number of people. The regeneration of osteochondral (OC) defects is one of the most challenging tasks in orthopedics since this anatomical region is composed of different tissues, characterized by antithetic features and functionalities, in tight connection to work together as a joint. The altered structural and mechanical joint environment impairs the natural tissue metabolism, thus making OC regeneration even more challenging. In this scenario, marine-derived ingredients elicit ever-increased interest for biomedical applications as a result of their outstanding mechanical and multiple biologic properties. The review highlights the possibility to exploit such unique features using a combination of bio-inspired synthesis process and 3D manufacturing technologies, relevant to generate compositionally and structurally graded hybrid constructs reproducing the smart architecture and biomechanical functions of natural OC regions.

## 1. State of the Art

The aging population, increasing obesity, and a surge in the number of sports-related injuries are the main drivers for the worldwide rising prevalence of bone and osteochondral (OC) diseases. Osteoarthritis (OA) refers to a rheumatic musculoskeletal disorder characterized by the progressive deterioration of the articular cartilage and sclerosis of the subchondral bone, together with changes in the synovial tissues, as a result of mechanical and biological processes that modify the cartilage homeostasis [1,2]. Injuries or repeated misaligned loading cause cartilage and subchondral bone damages, potentially contributing to the development of OA. An uncontrolled inflammatory response related to OA causes severe tissue damage and premature failure of any therapy. OA represents the most common complication in joint diseases and a leading cause of chronic disability, accounting for more than 25% of all new disability claims [3]. It was reported that 43% of people with OA are 65 or older and 88% of people with OA are 45 or older [4].

For several decades, both scientists and clinicians have been actively searching for an ideal cartilage implant that can reproduce the mechanical and biological properties of healthy, native cartilage. The inhibitory potential of some sulphated polysaccharides against various catabolic enzymes involved in joint degradation, such as hyaluronidases, elastases and matrix metalloproteases, was reported [5] and proposed as a new tool to prevent disease progression.

The altered joint environment and regenerative capacities make osteochondral regeneration even more challenging since this complex biologic process has to be sustained by a combination of mechanical and biological stimuli. Osteochondral defects affecting most of the condyle surface cannot yet be regenerated nor treated except using highly invasive approaches. Indeed, the implantation of metallic prosthesis is highly invasive and implies the ablation of a large amount of bone to assure the fixation. On the other hand, the existing regenerative devices have inadequate mechanical performance and insufficient bioactivity, hampering the re-growth of articular cartilage with a sliding surface and shock-absorption ability.

To overcome these bottlenecks, new approaches should be pursued that might be powered using novel smart materials and innovative engineering methods. In this respect, marine-derived materials are eliciting great interest as bioactive molecules or to develop scaffolds for biomedical applications such as wound healing and as drug delivery systems. Such an interest is raised by the outstanding performance of marine-derived tissues in terms of mechanical responsiveness and of multiple biologic abilities including anti-inflammatory, anticancer and antioxidant properties [5,6,7,8].

Boosted by such an interest, marine biomaterials experienced various stages of development, starting from coralline bone grafts and then moving to polysaccharide and protein-based biomaterials [9]. Relevant examples are sulphated polysaccharides such as glycosaminoglycans (GAGs), ulvan, carrageenan and fucoidan, showing outstanding biological properties and ability to activate biomineralization processes through interaction with proteins [10,11,12] as mediated by the degree of sulfation and negative charges [13].

Among proteins, collagen extracted from marine sources elicits great interest since it has an amino acid composition that is similar to human type I collagen [14]; in addition, its use can prevent cultural and sanitary concerns related to the use of terrestrial-derived collagen. For these reasons, the extraction of collagen from marine organisms such as sponges [15,16,17], fishes [18,19], squids [20] and echinoderms [21] is now regarded as the safest approach for large-scale collagen acquisition [22]. Similar to other natural polymers, marine-derived collagen can be processed using various forming technologies including 3D manufacturing and bioprinting to develop 2D and 3D constructs showing enhanced mechanical properties in association with a thinner fibrous structure compared to terrestrial animal-derived collagen [23,24]. In addition, marine-derived collagen can be conjugated with inorganic materials to obtain 3D multiphasic scaffolds with excellent osteogenesis ability [25].

Such considerations suggest that marine-derived materials are very promising compounds to be investigated for the development of multi-layered scaffolds for osteochondral regeneration. In this respect, the present review describes in more details the unique mechanical performance of the natural osteochondral unit and the relevance in reproducing such a performance to obtain effective osteochondral regeneration using scaffolds exhibiting high compositional and structural biomimicry. To this end, the development of multiphasic scaffolds is highlighted, as these are uniquely able to reproduce compositional and structural gradients typical of the different bony and cartilaginous compartments. On the other hand, the review focuses on the combination of bio-inspired mineralization processes and 3D manufacturing as promising technologies which might be synergistically combined to generate biomimetic multiphasic nanofibrillar structures, hierarchically organized from the nano to the macroscopic scale, as exhibited by the natural osteochondral unit.

## 2. Regenerative Approach: Effect of Mechanical Performance

Therapeutic interventions on damaged osteochondral regions should not only tackle cartilaginous damage, but the overall joint environment: indeed, the subchondral bone is always damaged in advanced OA and it may be further compromised by osteoporotic or inflammatory conditions, greatly influencing the course of the disease and treatment success. Until now, most attempts to regenerate osteochondral defects, particularly if affected by OA, have not resulted in a stable and reproducible restoration of joint function [26,27,28]. Various cell- and scaffold-based therapies are already available but only for focal cartilage and osteochondral defects [29,30,31]; however, cell therapies proved quite ineffective because, although there is evidence of transient chondrocyte proliferation at wound sites, there is little or no cellular migration into the injured sites to repair the defect [32,33]. On the other hand, most of the tested scaffolds showed insufficient bioactivity, resorption profile and timing not compliant with the rate of the regenerative process and, most importantly, ineffective mechanical performance to manage ever-changing, multi-axial forces [34,35,36]. Indeed, none of the present therapeutic approaches can consistently or completely restore the biomechanical properties of the complex osteochondral anatomical region [37]. Current surgical treatments for a full OC defect are limited and include microfracture or transplantation of OC tissue (allograft or autograft) [38,39]. While these treatments can provide some initial relief from pain, they have shown significant limitations, thus motivating research on regenerative approaches targeting full restoration of the damaged tissues as a long-term solution [40,41]. Similarly, mosaicplasty involves the transfer of OC cylindrical plugs from a non-load-bearing region of the patient’s joint to the defect [42,43,44,45,46]. While this treatment can relieve pain in many patients [47], the results are considerably less satisfactory when the defect is outside of a specified size range (typically 1–4 cm^2^ is recommended) or for patients over 35 [48,49,50,51,52]. Tissue engineering strategies aim to restore tissue function using methods which combine cells, tissue-inducing agents (such as growth factors) and scaffolds designed to guide regeneration [53].

To date, current cartilage scaffolds do not replicate the biomechanical performance of the natural osteochondral unit but mainly the ability to withstand compressive forces and to ensure sliding effects at the articular surface. These aspects are key in OC regeneration; however, most of the efforts in OC scaffold development have been focused only on the biological performance, leading to articular mechanical instability and failure.

The articular cartilage and its supporting subchondral bone represent a functional unit which is of paramount relevance for the joint mechanical performance [54]: the dynamic mechanical environment within the joint includes multi-axial compressive and shear strains as well as hydrostatic and osmotic pressures. The outstanding mechanical performance of the articular cartilage is given by both compositional and structural factors. In particular, the (hyaline) cartilage layer is prevalently composed of extracellular matrix consisting in water (70–80%), collagen (20–25%) and proteoglycans (PG), particularly aggrecans [55]. The latter act to collect and entrap water molecules inside the dense fiber network of the cartilage and have the function of expanding pressure, crucial for load distribution throughout its unique hierarchical structure and as a result of complex poro-viscoelastic properties. The smooth, lubricated cartilage surface (*lamina splendens*) accounts for extremely low friction and allows the transfer of multi-axial loads to the underlying subchondral bone, with the thin tidemark layer minimizing the stiffness gradient between the rigid bone and the more pliable cartilage [56]. Going from the surface to bone, the collagen fiber orientation progressively changes with depth from parallel to the surface towards perpendicular orientation, thus enabling proper anchoring to the underlying subchondral bone. In the same way, the concentration of proteoglycan aggregates (PG) increases with depth from the articular surface. This causes PG compaction under compressive strains of the cartilage, which increases the fixed charge density and water molecules’ linking. Therefore, the tissue permeability decreases, resulting in higher swelling pressures [57,58]. Such a phenomenon, known as fluid pressurization effect, is responsible for the high resistance to loading and excellent shock-absorption ability of the articular cartilage, thus being responsible for maintaining the integrity of the articular cartilage and protecting the underlying subchondral bone. The presence of the superficial cartilage layer known as *lamina splendens* represents a crucial element in the maintenance of virtually null friction conditions due to its unique ultrastructure and the constant production of molecules exerting a lubrication effect. Changes in the cartilage surface lead to the increase in the friction and result in the transformation of orthogonal into tangential forces with progressive deformation of the tissue architecture. In this respect, a major limitation in the existing cartilage repair devices is their inability to reproduce such a surface structure giving low friction conditions and correct load-dependent behavior, thus resulting in the progressive degeneration of the surrounding cartilage with time. The ensemble of the self-lubricating ability of the cartilage surface and the dynamically adaptive mechanisms, such as the fluid pressurization effect, are believed to be at the base of the unique shock-absorption properties of the articular cartilage and the key for the health of the entire osteochondral unit.

With this concept in mind, a general design that may drive the development of effective osteochondral scaffolds should recapitulate the composition and mechanically functional architecture of bony and cartilaginous tissues. This implies the realization of bioactive multiphase scaffolds, i.e., including both inorganic and organic compounds, engineered in such a way to reproduce the hybrid porous structure of the subchondral bone and the active mechanical responsiveness to multi-axial mechanical forces typical of the surmounting articular cartilage. In this respect, the outstanding physico-chemical, mechanical and biological properties of marine-derived compounds are very promising.

## 3. Multiphase Scaffolds

While there is no single material or fabrication method that has proven to be superior in developing an OC scaffold, a multiphase scaffold seems to be the best approach to creating tailored environments to regrow each of the tissue elements within the complex OC unit.

**Figure 1:**
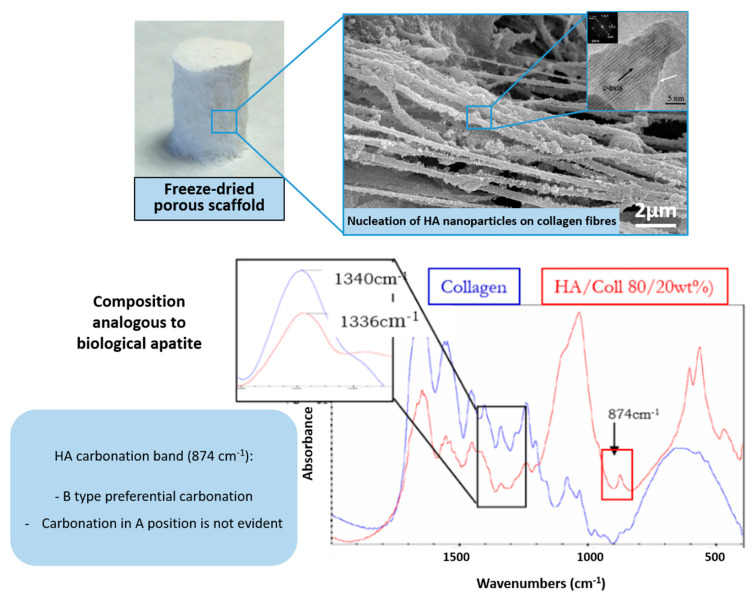
OC scaffold obtained via supramolecular organization and mineralization of collagen fibrils. (**Top**): macroscopic view of the scaffold (**left**) and microstructural details (**right**). (**Bottom**): TEM image showing the disordered state of the mineralizing apatitic phase (**left**) and FTIR spectrum showing the interaction between collagen and apatite related to heterogeneous nucleation (**right**).

To date, one of the most promising strategies to develop osteochondral substitutes comprises the generation of heterogeneous scaffolds that are obtained through the combination of distinct but integrated layers corresponding to the cartilage and bony regions. Such a design should be based on the recognition of the different requirements to regenerate the cartilaginous and bony parts of an osteochondral defect, and simultaneously prevent the risk of delamination of different components if these are adjacent but physically separated. To overcome this problem, a biomineralization process was previously developed, focusing on obtaining hybrid constructs through supramolecular assembling and mineralization of collagenous proteins, inspired by the natural phenomena occurring in the formation of several biologic structures such as shells, exoskeletons of marine animals and including bone and osteochondral regions [59]. The process, driven by appropriate temperature and pH conditions, was able to generate chemically and morphologically graded hybrid constructs mimicking the multi-tissue osteochondral region (Figure 1).

In detail, such a scaffold was built by stacking a bone-like mineralized layer produced according to the newly developed technique, an intermediate layer with a reduced amount of mineral phase to mimic the tidemark and an upper layer formed by collagen and hyaluronic acid, thus reproducing the cartilaginous environmental cues [59]. The biomineralization process was carried out in the presence of calcium, phosphate and other bioactive ions such as Mg^2+^ and CO_3_^2−^ to induce the heterogeneous nucleation of the apatitic phase on the collagen fibers during their assembling [60]. The resulting gel is subsequently treated via freeze-drying, forming the Mg-HA/Coll hybrid. From a bio-mechanical perspective, the final hybrid exhibited pseudo-plastic behavior, akin to bone tissue, and mechanical properties close to the values found for trabecular bone at corresponding values of porosity. Particularly, for porosity = 60%, Young modulus = 1.5–2 GPa and bending strength = 2–3 MPa; for scaffold porosity = 40%, Young modulus = 5–6 GPa and bending strength = 15 MPa [61].

In parallel, additive fabrication technologies have emerged in recent years, providing new tools for engineering living tissues, including bone and cartilage [62,63]. However, to date, these techniques have mainly been applied in the repair/regeneration of the cartilage, while defects greater than 6–8 mm in depth (OC defects) require the addition of a bone graft [38]. In this respect, approaches to obtain 3D printed multiphasic scaffold offer the potential to achieve improved results by repairing each of the native tissues in the OC unit. In fact, composition and structural gradients can be obtained using layer-by-layer fabrication processes, which also permit the deposition of polymeric and composite materials along organized 3D structures, attempting to mimic the osteochondral regions. A relevant advantage of 3D manufacturing is that these processes are guided by computer-aided design models, which can be directly obtained from CT scans of patient; thus, these approaches can drive the production of personalized scaffolds. 3D manufacturing also offers the possibility to precisely incorporate living cells, growth factors or other nanostructured components in well-defined geometries. It was shown, in this respect, that stem cells’ proliferation and differentiation can increase when in the presence of structurally anisotropic scaffolds [64].

Various 3D printing technologies are currently in development, and their full capabilities are an area for potential improvement in the design of biomimetic OC scaffolds (see Table 1 and Table 2). However, the selection of a fabrication method and the choice of suitable materials are interconnected and co-dependent steps within the OC scaffold design. For example, when selecting a material, its capability and compatibility in respect to the fabrication method should be taken into consideration (and vice versa).

In the native tissue, the articular cartilage is continuous, with an average pore size of only 6 nm; calcified cartilage has pores within the unmineralized areas which accounted for ≈22% of the tissue; and subchondral bone has an overall porosity of 64–94% [79,80,81]. A single fabrication method or a combination of different methods can be used to create the entire OC scaffold. The most widely used additive manufacturing techniques for creating 3D printed OC scaffolds include melt electro-writing (MEW), electrospinning (ESP), stereolithography and digital light processing (DLP) [82]. Recently emerged 3D printing processes include cryogenic 3D printing [83], powder-based printing [84,85], indirect printing [86,87,88], phase separation [89] and the use of custom-built printers [90]. Using a combination of 3D printing methods is a strategy that permits the combination of advantages and drawbacks of different techniques in order to better capture the varying tissue regions, especially in terms of mechanical functionality [91]. A smart example of processes combination was realized by H. Chen et al. [92] using direct writing (DW) and ESP to fabricate cartilage scaffolds that support cell seeding and proliferation. They fabricated a scaffold that recapitulates an arch-like structure, characterized by three of the four main zones of native cartilage: (i) the superficial zone, denoted by tangentially oriented collagen fibrils, was represented by two parallel electrospun layers with an average distance of about 200–300 µm; (ii) to reproduce the oblique collagen structure of the middle zone, crossed diagonal layers were created; finally, (iii) the deep zone was formed by parallel layers, radially oriented to the top structure. The reproduction of the natural *lamina splendens* with its sliding surface is relevant to achieve resistance to shear forces, a key aspect to improving the biomechanical performance of the scaffold under loading [93,94].

**Figure 2:**
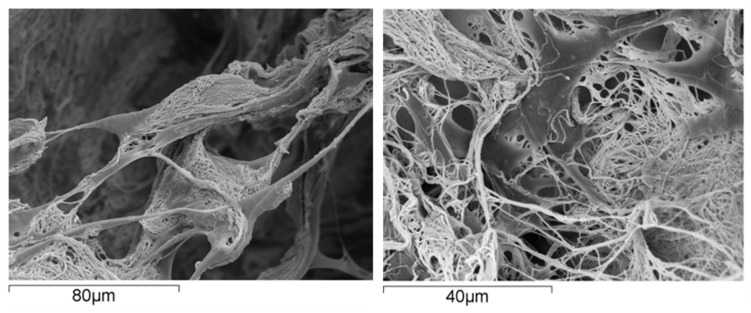
Human mesenchymal stem cells grown on a scaffold made of collagen fibrils; the cells exhibit excellent affinity with the substrate, as supported by their spread morphology and the presence of several arm-like cell projections, or pseudopodia, linking the cell to the substrate.

From the material perspective in OC scaffold development, natural polymers are commonly used in hydrogel form where their polymer networks are capable of holding a large amount of water, thereby creating a fully hydrated 3D environment comparable to that of the natural ECM [95,96]. This environment can support cell adhesion, proliferation of various cells and stimulate stem cell differentiation [97] (Figure 2).

**Figure 3:**
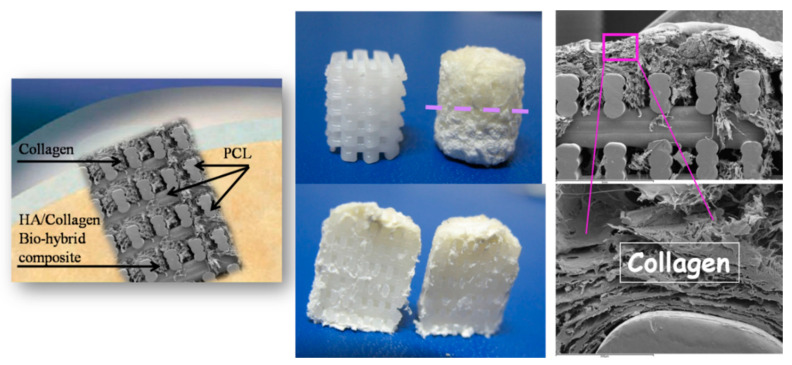
Scheme of the PCL scaffold filled with collagen in the cartilage-like part and with the collagen/apatite hybrid in the bone-like part. (**Left**): a pictorial image showing the implantation of the scaffold in the OC defect. Middle-up: the PCL matrix, as 3D printed (**left**) and filled with the hybrid (**right**): the dashed line highlights the interface between the bony and the cartilage-like layers. (**Right**): SEM images showing details of the scaffold with the filler.

On the other hand, natural polymers typically possess weak mechanical properties, which can lead to deformation in load-bearing areas [98]. Noticeably, these natural materials are commonly modified, especially using crosslinking (e.g., gelatin to gelatin methacryloyl (GelMA, hyaluronic acid to hyaluronic acid methacrylate) [99,100]. Conversely, the key advantages of synthetic polymers are enhanced mechanical properties (strength and stiffness) as well as their controllable biodegradability and processability [101]. The most commonly used polymers in OC scaffold manufacturing are based on polycaprolactone (PCL) due to its tunable biodegradability and approval by regulatory bodies [91,102,103,104,105,106,107,108,109,110], polylactic acid (PLA) and poly(L-lactic-co-glycolic acid) (PLGA) [89,106,111,112,113]. However, synthetic polymers, offering no specific biological signaling to cells [114], give rise to non-metabolic dissolution and negatively affect the regenerative cascade. A relevant example of such a drawback refers to previous experiments where mechanically reinforcing the spongy-like biomineralized Mg-HA/Coll hybrid (Mg-HA nucleated on collagen as above described) was attempted using a 3D printed network of PCL, in turn loaded with 30% of HA phase (Figure 3).

**Figure 4:**
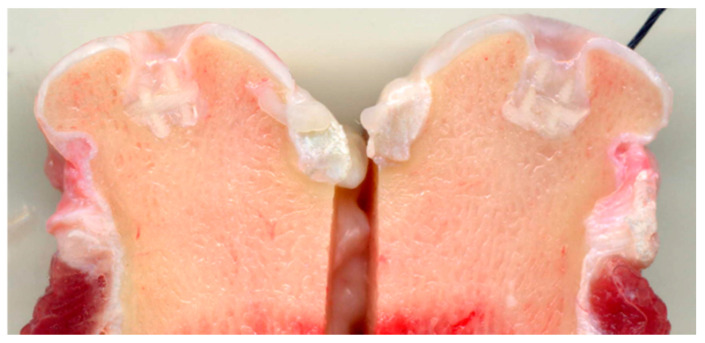
Bone sheep knee explants at 3 months after implantation of PCL scaffolds filled with collagen/apatite hybrid matrix. The picture clearly shows the presence of fibrous tissue in the OC defects, attributed to the presence of extensive amount of PCL in the scaffold.

The surface wettability of the polymeric network is strongly increased by the presence of biomimetic HA powder inside and on the surface of PCL pillars, thus favoring cell adhesion and colonization. The final scaffold was obtained by mimicking the three osteochondral layers as above described and infiltrating them inside the macro-porous PCL structure. The final scaffolds were implanted in critical knee osteochondral defects created in a group of 6 sheep. In spite of the presence of the Mg-HA/Col hybrid filling all the space inside the polymeric network, very promising to enhancing the bioactivity of the scaffold [115], abundant amount of fibrous tissue was generated after 3 months (Figure 4), thus confirming the adverse effects caused by the dissolution of the synthetic polymer, affecting cells differentiation particularly in closed defects characterized by low levels of fluids exchange. A complete absence of bone and cartilage regeneration was observed in all implants. Despite the lack of regeneration and presence of fibrous tissue, there were no osteolytic changes around the implant, which are frequently observed with PCL implants, probably due to the presence of biomimetic HA coating.

Additionally relevant is polyethylene glycol (PEG), which is primarily used in the cartilage region of the scaffold [109,116,117,118]. Hydroxyapatite and tricalcium phosphate in form of cements, pastes or 3D scaffold are the most commonly used bioceramics for the subchondral bone regeneration [110,116,119,120,121,122,123]. However, these materials naturally exist as brittle powders, thereby limiting their ability to form free-standing porous structures on their own [124].

## 4. Marine Ingredients in the Development of OC Scaffolds: Future Perspectives

In recent decades, the extrusion technology has been widely implemented to develop solutions for tissue engineering [125,126].

Considering the advantages and drawbacks related to different elective approaches for OC scaffold development, the association of bio-inspired assembling/mineralization processes and 3D fabrication techniques is a promising approach for a leap forward in the development of multi-layered scaffolds suitable for OC tissue regeneration.

It is well-known that porosity, particularly when organized in hierarchical architectures, greatly influences the mechanical properties with reference to viscoelastic deformation in hybrid scaffolds. However, to achieve the cell chemotaxis and thus activate appropriate cell behavior yielding regeneration of healthy OC region with its outstanding mechanical performance, the chemical composition and scaffold biomimicry are also a key aspect. In fact, the appropriate choice of materials for OC scaffold permits the tuning of the bioactivity by exploiting biomolecular recognition by stem cells and chondrocytes; at the same time, however, the functional structural arrangement of constituting elements is a major aspect determining the mechanical performance of the regenerated OC region. To implement an appropriate synergy between chemistry and 3D architecture in OC scaffolds, marine-derived materials can be a relevant source of inspiration, particularly echinoderms endowed with smart connective tissues made of mutable collagenous proteins (MCP) able to dynamically modify the stiffness of the extracellular matrix. The biomimetic composition and structure could provide the OC scaffold with a high regenerative ability and mechanical competence as a result of the activation of chemical mechanisms modulating the interfibrillar cohesion and the elastic–plastic stress transfer dependent on the strength of the applied load [127,128]. As a result of the inherent bioactivity of marine-derived compounds, their use as base materials for OC scaffold can be opened up to unprecedented biologic performances, as these are able to elicit and sustain the regeneration of both articular cartilage and the underlying bony tissues, in addition to being promising in the presence of impairing physio-pathological conditions such as osteoporosis, low endogenous potential—typical of the elderly—chronic inflammatory states, or metabolic syndrome-associated osteoarthritis. Inspired by previous results, hybrid bony layers may be obtained through the mineralization of MCP with biomimetic mineral phases such as ion-doped nano-hydroxyapatite. As a result of their similarity with the natural mineral bone, apatites can assure osteoblastic differentiation; the presence of Mg^2+^, substituting Ca^2+^ in dynamic equilibrium with the physiological environment, is known to stimulate the regenerative process, while the presence of Sr^2+^ affects the osteoblast–osteoclast cross-talk favoring the osteoblast activity, chiefly relevant to contrast osteoporosis [129,130]. Besides the chemical mimesis with natural tissues, the use of fibrous hybrid constructs as scaffolds for bone regeneration is interesting as they can increase bio-stimulating piezoelectric currents during the natural body movement, as occurring in the natural bone [131], due to the different stiffness of the mineral phases and collagen fibrils forming the hybrid, as well as their mutual sliding.

For the cartilaginous layer, the use of MCP, prevalently formed of marine-derived mutable collagen, can be effective to achieve an enhanced mechanical performance. Indeed, contrary to mammalian-derived collagen, MCP does not require harsh processes for the extraction and purification of fibrils [132]. In fact, it is relatively easy to obtain a high amount of native collagen fibrils from echinoderms using non-denaturating chemical processes as a result of the relatively weak nature of the interactions that maintain interfibrillar cohesion in MCP. This allows the retention of the original fibril ultrastructure, tensile strength and stiffness, thus facilitating the assembly of MCP fibrils into 3D cartilage-like structures with enhanced mechanical performance. In fact, echinoderm-derived collagen is characterized by thinner fibrils, organized in a random fashion and as forming a more compact structure in comparison with bovine collagen [23]. This is associated with significantly higher stiffness and tensile strength (~20 folds), particularly collagen derived from sea cucumber. Previous investigation demonstrated that the beaded micro-fibrils are ubiquitous in MCP and consists at least partly of fibrillin-like protein. These micro-fibrils may facilitate slippage between adjacent fibrils bundles during MCP deformation and contribute to passive elastic recoil after the removal of external forces [133]. To further increase the fibration ability and mechanical performance, MCP can be intercalated with animal-derived collagen characterized by long-chain molecules, such as type I collagen derived from equine tendon. Equine tendons possess a hierarchical organization in comparison with other collagen sources [134]. Further, its peculiar amino acid composition and stronger fibers packing make native horse tendon collagen intrinsically more resistant to degradation [135,136].

In association with MCP, marine-derived polysaccharides such as fucoidan (FUC) elicit great interest as a result of their excellent bioactivity and unique structure. Given the presence of negatively charged functional groups, FUC can be chemically linked to MCP by activating reactions between the sulfate group and free amines; this is suitable to achieve stronger inter-fibrillary interactions and marked increase in the compressive strength. This can be relevant to dynamically regulate interfibrillar shear stress and the fibril–fibril sliding under loading, in turn affecting the elastic–plastic stress transfer [7]. As FUC has also great affinity with water and proteins, the extent of crosslinking is also relevant to tune the water adsorption ability [137], in turn influencing the extent of water exudation and the derived fluid pressurization effect under compressive loading. FUC also plays a relevant role in bone morphogenesis since it has been demonstrated to interact with transforming growth factor (TGF)-β1, facilitating bone-like apatite formation and the expression of osteocalcin, whilst supporting human bone marrow stromal cells (hBMSC) growth and chondrocytic differentiation. In this respect, it was found that fucoidan stimulates the production of GAGs as a marker of mature chondrocyte phenotype [138]. In addition to tissue-specific effects, FUC plays an important role also in several biological functions including immunomodulatory, antibacterial, anti-inflammatory and antioxidant effects [6]. Indeed, recent studies demonstrated that fucoidans are capable of inhibiting histamine release, protein kinases and COX-2 [139]. In particular, the enzyme inhibitory potential of fucoidans against hyaluronidases, elastases and matrix metalloproteases (MMPs) has been well-documented [5] and can be used to contrast the inflammation of matrices mainly composed of collagens and hyaluronan such as cartilage. Indeed, MMPs are associated with the degradation and remodeling of extracellular matrix components, as documented in experiments with human primary chondrocytes and primary culture of bovine chondrocytes [8,140].

**Figure 5:**
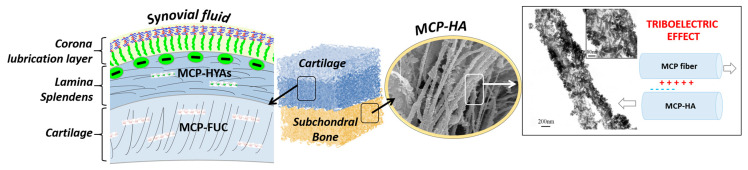
Schematic representation of a possible approach for OC scaffold development using marine-derived ingredients, from left to right. In particular, mutable collagen proteins (MCP) are characterized by adaptive interfibrillar cohesion, thus exhibiting mechanical competence and high regenerative ability. The non-mineralized cartilage-like layer, indicated as “cartilage”, can be obtained by functionalizing MCP matrix with fucoidans (FUC), capable to coordinate water molecules. Conversely, the subchondral bone layer can be obtained by MCP mineralized with ion-doped apatitic phases, thus acting as bioactive chemical signals for stem cells differentiation, as well as promoting the rising of bio-stimulating piezo/tribo-electric effect, as occurs in the natural osteochondral region.

In consideration of the above-reported, an interesting future perspective in the development of OC scaffolds with enhanced regenerative ability is given by the convergence of novel 3D fabrication technologies with materials endowed with unique bioactive and structural properties, such as marine-derived proteins and polysaccharides. Such an approach is promising as regards important advances in terms of still unmet mechanical and biologic performance of scaffolds for regenerative medicine, particularly addressing the regeneration of large, load-bearing OC defects (Figure 5).

## 5. Conclusions

The present review focused on osteochondral regeneration as a still unmet clinical need from a material science perspective. In this respect, we investigated the existing literature, highlighting the most important aspects relevant for the achievement of clinically effective scaffold-based solutions. We thus highlighted the relevance of using marine-derived ingredients given their outstanding mechanic and biologic properties as a new concept guiding material scientists in the development of osteochondral scaffolds.

In particular, we presented a rationale sustaining the combination of bio-inspired synthesis methods with advanced 3D manufacturing technologies, as a synergistic approach allowing the translation of the unique biomechanical and regenerative performances of marine-derived ingredients into new bioactive multiphase scaffolds for osteochondral regeneration. Firstly, mutable collagen proteins (MCP), characterized by adaptive interfibrillar cohesion, can be explored for their mechanical competence and high regenerative ability. In particular, MCP can be assembled and mineralized using ion-doped apatitic phases acting as bioactive chemical signals for stem cells, as well as promoting the rising of bio-stimulating piezo/tribo-electric currents, as occurs in the natural osteochondral region. On the other hand, the non-mineralized cartilage-like layer can be obtained by functionalizing MCP matrix with fucoidans, which are able to entrap water molecules and cross-link the MCP matrix, responding to applied mechanical loads. This ability is promising and represents the missing piece to reproduce the fluid pressurization effect, which is a key mechanism in ensuring the mechanical integrity and the health of the whole osteochondral unit.

Finally, we highlighted the synergistic use of 3D manufacturing technologies to generate multi-scale, mechanically competent arch-like architectures able to cope with multi-axial compressive shocks. In this respect, electrospinning and electrowriting techniques are techniques of particular interest as they can be implemented to generate wear-free surfaces mimicking the *lamina splendens*, and are able to prevent cartilage disruption caused by shear forces. Such a challenging achievement is considered a cornerstone for the development of osteochondral scaffolds effective for the regeneration of large, load-bearing defects.

Importantly, the inherent anti-inflammatory and antioxidant properties of marine-derived ingredients are of great interest to contrast osteoarthritis and osteoporosis-related degenerative diseases.

## Figures and Tables

**Table 1:** Marine-derived bio-inks for bone tissue engineering (extrusion bioprinting technology).

Marine-Derived Biomaterial/Marine Resource	Bioink Composites	References
Alginate/Brown Algae	Alginate/multipotent stromal cells	[65]
	Alginate/silica/biosilica/SaOS-2 cells	[66]
	Wood-based cellulose nanofibrils/bioactive glass/gelatin/alginate/Saos-2 cells/hMSCs	[67]
Carrageenan/Red Algae	k-Carrageenan/CaP pastes	[68]
Chitosan/Shell	Chitosan/hydroxyapatite/MC3T3 mouse preosteoblasts	[69]

**Table 2:** Marine-derived bio-inks for cartilage tissue engineering (extrusion bioprinting technology).

Marine-Derived Biomaterial/Marine Resource	Bioink Composites	References
Alginate/Brown Algae	Alginate/osteoblasts/chondrocytes	[70]
	Alginate/CPCs	[71]
	Cartilage decellularized extracellular matrix/alginate/hMSCs	[72]
	Hydroxyapatite/alginate/chondrocyte	[73]
Carrageenan/Red Algae	MA-k-carrageenan/2D nanosilicates/hMSC	[74]
	k-Carrageenan/Nanosilicates (k-CAR/nSi)	[75]
	Methacrylamide-modified gelatin (GELMA)/methacrylated k-CAR (CARMA)	[76]
Chitosan/Shell	Chitosan/oxidized chondroitin sulfate/hADMSCs	[77]
	Hyaluronate/chitosan/adipic acid dihydrazide/ATDC5 chondrocyte	[78]

## Data Availability

No new data were created/described in this paper.

## References

[1] Pritzker K.P.H., Gay S., Jimenez S.A., Ostergaard K., Pelletier J.P., Revell P.A., Salter D., van den Berg W.B. (2006). Osteoarthritis cartilage histopathology: Grading and staging. Osteoarthr. Cartil..

[2] Vos T., Flaxman A.D., Naghavi M., Lozano R., Michaud C., Ezzati M., Shibuya K., Salomon J.A., Abdalla S., Aboyans V. (2012). Years lived with disability (YLDs) for 1160 sequelae of 289 diseases and injuries 1990-2010: A systematic analysis for the Global Burden of Disease Study 2010. Lancet.

[3] Messier S.P., Ettinger W.H., Doyle T.E., Morgan T., James M.K., O’Toole M.L., Burns R. (1996). Obesity: Effects on Gait in an Osteoarthritic Population. J. Appl. Biomech..

[4] United States Bone and Joint Initiative The Burden of Musculoskeletal Diseases in the United States (BMUS). https://www.boneandjointburden.org/.

[5] Fitton J.H., Stringer D.N., Karpiniec S.S. (2015). Therapies from Fucoidan: An Update. Mar. Drugs.

[6] Jensen G.S., Lenninger M.R., Beaman J.L., Taylor R., Benson K.F. (2015). Support of Joint Function, Range of Motion, and Physical Activity Levels by Consumption of a Water-Soluble Egg Membrane Hydrolyzate. J. Med. Food.

[7] Mo J., Prevost S.F., Blowes L.M., Egertova M., Terrill N.J., Wang W., Elphick M.R., Gupta H.S. (2016). Interfibrillar stiffening of echinoderm mutable collagenous tissue demonstrated at the nanoscale. Proc. Natl. Acad. Sci. USA.

[8] Phull A.R., Majid M., Haq I.U., Khan M.R., Kim S.J. (2017). In vitro and in vivo evaluation of anti-arthritic, antioxidant efficacy of fucoidan from Undaria pinnatifida (Harvey) Suringar. Int. J. Biol. Macromol..

[9] Wan M.-c., Qin W., Lei C., Li Q.-h., Meng M., Fang M., Song W., Chen J.-h., Tay F., Niu L.-n. (2021). Biomaterials from the sea: Future building blocks for biomedical applications. Bioact. Mater..

[10] Cui J., Li Y., Wang S., Chi Y., Hwang H., Wang P. (2018). Directional preparation of anticoagulant-active sulfated polysaccharides from Enteromorpha prolifera using artificial neural networks. Sci. Rep..

[11] Gao Y., Zhang L., Jiao W., Zhang L. (2019). Chapter Seven—Marine glycan-derived therapeutics in China. Progress in Molecular Biology and Translational Science.

[12] Venkatesan J., Anil S., Rao S., Bhatnagar I., Kim S.-K. (2019). Sulfated Polysaccharides from Macroalgae for Bone Tissue Regeneration. Curr. Pharm. Des..

[13] Costa D.S.d., Reis R.L., Pashkuleva I. (2017). Sulfation of Glycosaminoglycans and Its Implications in Human Health and Disorders. Annu. Rev. Biomed. Eng..

[14] Silva J.C., Barros A.A., Aroso I.M., Fassini D., Silva T.H., Reis R.L., Duarte A.R.C. (2016). Extraction of Collagen/Gelatin from the Marine Demosponge Chondrosia reniformis (Nardo, 1847) Using Water Acidified with Carbon Dioxide—Process Optimization. Ind. Eng. Chem. Res..

[15] Barros A.A., Aroso I.M., Silva T.H., Mano J.F., Duarte A.R.C., Reis R.L. (2015). Water and Carbon Dioxide: Green Solvents for the Extraction of Collagen/Gelatin from Marine Sponges. ACS Sustain. Chem. Eng..

[16] Swatschek D., Schatton W., Kellermann J., Müller W.E.G., Kreuter J. (2002). Marine sponge collagen: Isolation, characterization and effects on the skin parameters surface-pH, moisture and sebum. Eur. J. Pharm. Biopharm..

[17] Zheng M.H., Hinterkeuser K., Solomon K., Kunert V., Pavlos N.J., Xu J. (2007). Collagen-Derived Biomaterials in Bone and Cartilage Repair. Macromol. Symp..

[18] Mahboob S. (2015). Isolation and characterization of collagen from fish waste material- skin, scales and fins of Catla catla and Cirrhinus mrigala. J. Food Sci. Technol..

[19] Nagai N., Yunoki S., Suzuki T., Sakata M., Tajima K., Munekata M. (2004). Application of cross-linked salmon atelocollagen to the scaffold of human periodontal ligament cells. J. Biosci. Bioeng..

[20] Coelho R.C.G., Marques A.L.P., Oliveira S.M., Diogo G.S., Pirraco R.P., Moreira-Silva J., Xavier J.C., Reis R.L., Silva T.H., Mano J.F. (2017). Extraction and characterization of collagen from Antarctic and Sub-Antarctic squid and its potential application in hybrid scaffolds for tissue engineering. Mater. Sci. Eng. C.

[21] Khattak S., Wahid F., Liu L.-P., Jia S.-R., Chu L.-Q., Xie Y.-Y., Li Z.-X., Zhong C. (2019). Applications of cellulose and chitin/chitosan derivatives and composites as antibacterial materials: Current state and perspectives. Appl. Microbiol. Biotechnol..

[22] Pal G.K., Suresh P.V. (2016). Sustainable valorisation of seafood by-products: Recovery of collagen and development of collagen-based novel functional food ingredients. Innov. Food Sci. Emerg. Technol..

[23] Ferrario C., Leggio L., Leone R., Di Benedetto C., Guidetti L., Coccè V., Ascagni M., Bonasoro F., La Porta C.A.M., Candia Carnevali M.D. (2017). Marine-derived collagen biomaterials from echinoderm connective tissues. Mar. Environ. Res..

[24] Gopinathan J., Noh I. (2018). Recent trends in bioinks for 3D printing. Biomater. Res..

[25] Diogo G.S., López-Senra E., Pirraco R.P., Canadas R.F., Fernandes E.M., Serra J., Pérez-Martín R.I., Sotelo C.G., Marques A.P., González P. (2018). Marine collagen/apatite composite scaffolds envisaging hard tissue applications. Mar. Drugs.

[26] Henderson I., Lavigne P., Valenzuela H., Oakes B. (2007). Autologous chondrocyte implantation: Superior biologic properties of hyaline cartilage repairs. Clin. Orthop. Relat. Res..

[27] Knutsen G., Drogset J.O., Engebretsen L., Grøntvedt T., Ludvigsen T.C., Løken S., Solheim E., Strand T., Johansen O. (2016). A Randomized Multicenter Trial Comparing Autologous Chondrocyte Implantation with Microfracture: Long-Term Follow-up at 14 to 15 Years. J. Bone Jt. Surg. Am..

[28] Kraeutler M.J., Aberle N.S., Brown C.C., Ptasinski J.J., McCarty E.C. (2018). Clinical Outcomes and Return to Sport After Arthroscopic Anterior, Posterior, and Combined Shoulder Stabilization. Orthop. J. Sports Med..

[29] Knutsen G., Engebretsen L., Ludvigsen T.C., Drogset J.O., Grontvedt T., Solheim E., Strand T., Roberts S., Isaksen V., Johansen O. (2004). Autologous chondrocyte implantation compared with microfracture in the knee. A randomized trial. J. Bone Jt. Surg. Am..

[30] Niemeyer P., Albrecht D., Andereya S., Angele P., Ateschrang A., Aurich M., Baumann M., Bosch U., Erggelet C., Fickert S. (2016). Autologous chondrocyte implantation (ACI) for cartilage defects of the knee: A guideline by the working group “Clinical Tissue Regeneration” of the German Society of Orthopaedics and Trauma (DGOU). Knee.

[31] Zhang W., Ouyang H., Dass C.R., Xu J. (2016). Current research on pharmacologic and regenerative therapies for osteoarthritis. Bone Res..

[32] Brittberg M., Lindahl A., Nilsson A., Ohlsson C., Isaksson O., Peterson L. (1994). Treatment of Deep Cartilage Defects in the Knee with Autologous Chondrocyte Transplantation. N. Engl. J. Med..

[33] Levingstone T.J., Moran C., Almeida H.V., Kelly D.J., O’Brien F.J. (2021). Layer-specific stem cell differentiation in tri-layered tissue engineering biomaterials: Towards development of a single-stage cell-based approach for osteochondral defect repair. Mater. Today Bio.

[34] Deng C., Chang J., Wu C. (2019). Bioactive scaffolds for osteochondral regeneration. J. Orthop. Transl..

[35] Tamaddon M., Gilja H., Wang L., Oliveira J.M., Sun X., Tan R., Liu C. (2020). Osteochondral scaffolds for early treatment of cartilage defects in osteoarthritic joints: From bench to clinic. Biomater. Transl..

[36] Zhang B., Huang J., Narayan R.J. (2020). Gradient scaffolds for osteochondral tissue engineering and regeneration. J. Mater. Chem. B.

[37] Jayasuriya C.T., Twomey-Kozak J., Newberry J., Desai S., Feltman P., Franco J.R., Li N., Terek R., Ehrlich M.G., Owens B.D. (2019). Human Cartilage-Derived Progenitors Resist Terminal Differentiation and Require CXCR4 Activation to Successfully Bridge Meniscus Tissue Tears. Stem Cells.

[38] Burns T.C., Giuliani J.R., Svoboda S.J., Owens B.D. (2010). Knee Cartilage Tibio-Femoral Injuries. Tech. Orthop..

[39] Salzmann G.M., Niemeyer P., Hochrein A., Stoddart M.J., Angele P. (2018). Articular Cartilage Repair of the Knee in Children and Adolescents. Orthop. J. Sports Med..

[40] Lepage S.I.M., Robson N., Gilmore H., Davis O., Hooper A., St John S., Kamesan V., Gelis P., Carvajal D., Hurtig M. (2019). Beyond Cartilage Repair: The Role of the Osteochondral Unit in Joint Health and Disease. Tissue Eng. Part. B Rev..

[41] Ricci M., Tradati D., Maione A., Uboldi F.M., Usellini E., Berruto M. (2021). Cell-free osteochondral scaffolds provide a substantial clinical benefit in the treatment of osteochondral defects at a minimum follow-up of 5 years. J. Exp. Orthop..

[42] Bader S., Miniaci A. (2011). Mosaïcplasty. Orthopedics.

[43] Robert H. (2011). Chondral repair of the knee joint using mosaicplasty. Orthop. Traumatol. Surg. Res..

[44] Robinson P.G., Williamson T., Murray I.R., Al-Hourani K., White T.O. (2020). Sporting participation following the operative management of chondral defects of the knee at mid-term follow up: A systematic review and meta-analysis. J. Exp. Orthop..

[45] Rowland R., Colello M., Wyland D.J. (2019). Osteochondral Autograft Transfer Procedure: Arthroscopic Technique and Technical Pearls. Arthrosc. Tech..

[46] Shekhar A., Reddy S., Patil S., Tapasvi S. (2021). Mid-term outcomes of arthroscopic osteochondral autograft transplantation for focal chondral defects of the knee. J. Arthrosc. Surg. Sports Med..

[47] Pareek A., Reardon P.J., Maak T.G., Levy B.A., Stuart M.J., Krych A.J. (2016). Long-term Outcomes After Osteochondral Autograft Transfer: A Systematic Review at Mean Follow-up of 10.2 Years. Arthrosc. J. Arthrosc. Relat. Surg..

[48] Hangody L., Füles P. (2003). Autologous Osteochondral Mosaicplasty for the Treatment of Full-Thickness Defects of Weight-Bearing Joints: Ten Years of Experimental and Clinical Experience. J. Bone Joint Surg. Am..

[49] Ma H.L., Hung S.C., Wang S.T., Chang M.C., Chen T.H. (2004). Osteochondral autografts transfer for post-traumatic osteochondral defect of the knee-2 to 5 years follow-up. Injury.

[50] Richter D.L., Schenck R.C., Wascher D.C., Treme G. (2015). Knee Articular Cartilage Repair and Restoration Techniques: A Review of the Literature. Sports Health.

[51] Solheim E., Hegna J., Øyen J., Harlem T., Strand T. (2013). Results at 10 to 14 years after osteochondral autografting (mosaicplasty) in articular cartilage defects in the knee. Knee.

[52] Stone A.V., Christian D.R., Redondo M.L., Yanke A.B., Southworth T.M., Tauro T.M., Cole B.J. (2018). Osteochondral Allograft Transplantation and Osteochondral Autograft Transfer. Oper. Tech. Sports Med..

[53] Langer R., Vacanti J.P. (1993). Tissue Engineering. Science.

[54] Mow V.C., Guo X.E. (2002). Mechano-electrochemical properties of articular cartilage: Their inhomogeneities and anisotropies. Annu. Rev. Biomed. Eng..

[55] Osaki T., Kitahara K., Okamoto Y., Imagawa T., Tsuka T., Miki Y., Kawamoto H., Saimoto H., Minami S. (2012). Effect of fucoidan extracted from mozuku on experimental cartilaginous tissue injury. Mar. Drugs.

[56] Redler I., Mow V.C., Zimny M.L., Mansell J. (1975). The ultrastructure and biomechanical significance of the tidemark of articular cartilage. Clin. Orthop. Relat. Res..

[57] Jung E., Choi H.J., Lim M., Kang H., Park H., Han H., Min B.-h., Kim S., Park I., Lim H. (2012). Quantitative analysis of water distribution in human articular cartilage using terahertz time-domain spectroscopy. Biomed. Opt. Express.

[58] Maroudas A. (1976). Balance between swelling pressure and collagen tension in normal and degenerate cartilage. Nature.

[59] Tampieri A., Sandri M., Landi E., Pressato D., Francioli S., Quarto R., Martin I. (2008). Design of graded biomimetic osteochondral composite scaffolds. Biomaterials.

[60] LeGeros R.Z. (1991). Calcium phosphates in oral biology and medicine. Monogr. Oral Sci..

[61] Tampieri A., Celotti G., Landi E. (2005). From biomimetic apatites to biologically inspired composites. Anal. Bioanal. Chem..

[62] Abdollahiyan P., Oroojalian F., Mokhtarzadeh A., de la Guardia M. (2020). Hydrogel-Based 3D Bioprinting for Bone and Cartilage Tissue Engineering. Biotechnol. J..

[63] Harley W.S., Li C.C., Toombs J., O’Connell C.D., Taylor H.K., Heath D.E., Collins D.J. (2021). Advances in biofabrication techniques towards functional bioprinted heterogeneous engineered tissues: A comprehensive review. Bioprinting.

[64] Nowicki M.A., Castro N.J., Plesniak M.W., Zhang L.G. (2016). 3D printing of novel osteochondral scaffolds with graded microstructure. Nanotechnology.

[65] Loozen L.D., Wegman F., Öner F.C., Dhert W.J.A., Alblas J. (2013). Porous bioprinted constructs in BMP-2 non-viral gene therapy for bone tissue engineering. J. Mater. Chem. B.

[66] Müller W.E.G., Schröder H.C., Feng Q., Schlossmacher U., Link T., Wang X. (2015). Development of a morphogenetically active scaffold for three-dimensional growth of bone cells: Biosilica–alginate hydrogel for SaOS-2 cell cultivation. J. Tissue Eng. Regen. Med..

[67] Law N., Doney B., Glover H., Qin Y., Aman Z.M., Sercombe T.B., Liew L.J., Dilley R.J., Doyle B.J. (2018). Characterisation of hyaluronic acid methylcellulose hydrogels for 3D bioprinting. J. Mech. Behav. Biomed. Mater..

[68] Kelder C., Bakker A.D., Klein-Nulend J., Wismeijer D. (2018). The 3D Printing of Calcium Phosphate with K-Carrageenan under Conditions Permitting the Incorporation of Biological Components-A Method. J. Funct. Biomater..

[69] Demirtaş T.T., Irmak G., Gümüşderelioğlu M. (2017). A bioprintable form of chitosan hydrogel for bone tissue engineering. Biofabrication.

[70] Shim J.-H., Lee J.-S., Kim J.Y., Cho D.-W. (2012). Bioprinting of a mechanically enhanced three-dimensional dual cell-laden construct for osteochondral tissue engineering using a multi-head tissue/organ building system. J. Micromech. Microeng..

[71] Zhang Y., Yu Y., Chen H., Ozbolat I.T. (2013). Characterization of printable cellular micro-fluidic channels for tissue engineering. Biofabrication.

[72] Rathan S., Dejob L., Schipani R., Haffner B., Möbius M.E., Kelly D.J. (2019). Fiber Reinforced Cartilage ECM Functionalized Bioinks for Functional Cartilage Tissue Engineering. Adv. Heal. Mater..

[73] You F., Chen X., Cooper D.M.L., Chang T., Eames B.F. (2019). Homogeneous hydroxyapatite/alginate composite hydrogel promotes calcified cartilage matrix deposition with potential for three-dimensional bioprinting. Biofabrication.

[74] Thakur A., Jaiswal M.K., Peak C.W., Carrow J.K., Gentry J., Dolatshahi-Pirouz A., Gaharwar A.K. (2016). Injectable shear-thinning nanoengineered hydrogels for stem cell delivery. Nanoscale.

[75] Wilson S.A., Cross L.M., Peak C.W., Gaharwar A.K. (2017). Shear-Thinning and Thermo-Reversible Nanoengineered Inks for 3D Bioprinting. ACS Appl. Mater. Interfaces.

[76] Tytgat L., Van Damme L., Ortega Arevalo M.D.P., Declercq H., Thienpont H., Otteveare H., Blondeel P., Dubruel P., Van Vlierberghe S. (2019). Extrusion-based 3D printing of photo-crosslinkable gelatin and κ-carrageenan hydrogel blends for adipose tissue regeneration. Int. J. Biol. Macromol..

[77] Li C., Wang K., Zhou X., Li T., Xu Y., Qiang L., Peng M., Xu Y., Xie L., He C. (2019). Controllable fabrication of hydroxybutyl chitosan/oxidized chondroitin sulfate hydrogels by 3D bioprinting technique for cartilage tissue engineering. Biomed. Mater..

[78] Kim S.W., Kim D.Y., Roh H.H., Kim H.S., Lee J.W., Lee K.Y. (2019). Three-Dimensional Bioprinting of Cell-Laden Constructs Using Polysaccharide-Based Self-Healing Hydrogels. Biomacromolecules.

[79] Di Luca A., Van Blitterswijk C., Moroni L. (2015). The osteochondral interface as a gradient tissue: From development to the fabrication of gradient scaffolds for regenerative medicine. Birth Defects Res. Part C.

[80] Fan X., Wu X., Crawford R., Xiao Y., Prasadam I. (2021). Macro, Micro, and Molecular. Changes of the Osteochondral Interface in Osteoarthritis Development. Front. Cell Dev. Biol..

[81] Mow V.C., Holmes M.H., Michael Lai W. (1984). Fluid transport and mechanical properties of articular cartilage: A review. J. Biomech..

[82] Zhu W., Ma X., Gou M., Mei D., Zhang K., Chen S. (2016). 3D printing of functional biomaterials for tissue engineering. Curr. Opin. Biotechnol..

[83] Adamkiewicz M., Rubinsky B. (2015). Cryogenic 3D printing for tissue engineering. Cryobiology.

[84] Du Y., Liu H., Yang Q., Wang S., Wang J., Ma J., Noh I., Mikos A.G., Zhang S. (2017). Selective laser sintering scaffold with hierarchical architecture and gradient composition for osteochondral repair in rabbits. Biomaterials.

[85] Zhou Z., Buchanan F., Mitchell C., Dunne N. (2014). Printability of calcium phosphate: Calcium sulfate powders for the application of tissue engineered bone scaffolds using the 3D printing technique. Mater. Sci. Eng. C.

[86] Castro N.J., Patel R., Zhang L.G. (2015). Design of a Novel 3D Printed Bioactive Nanocomposite Scaffold for Improved Osteochondral Regeneration. Cell. Mol. Bioeng..

[87] Lee J.Y., Choi B., Wu B., Lee M. (2013). Customized biomimetic scaffolds created by indirect three-dimensional printing for tissue engineering. Biofabrication.

[88] Nowicki M., Zhu W., Sarkar K., Rao R., Zhang L.G. (2020). 3D printing multiphasic osteochondral tissue constructs with nano to micro features via PCL based bioink. Bioprinting.

[89] Zhang T., Zhang H., Zhang L., Jia S., Liu J., Xiong Z., Sun W. (2017). Biomimetic design and fabrication of multilayered osteochondral scaffolds by low-temperature deposition manufacturing and thermal-induced phase-separation techniques. Biofabrication.

[90] Li Z., Jia S., Xiong Z., Long Q., Yan S., Hao F., Liu J., Yuan Z. (2018). 3D-printed scaffolds with calcified layer for osteochondral tissue engineering. J. Biosci. Bioeng..

[91] Diloksumpan P., de Ruijter M., Castilho M., Gbureck U., Vermonden T., van Weeren P.R., Malda J., Levato R. (2020). Combining multi-scale 3D printing technologies to engineer reinforced hydrogel-ceramic interfaces. Biofabrication.

[92] Chen H., Malheiro A., van Blitterswijk C., Mota C., Wieringa P.A., Moroni L. (2017). Direct Writing Electrospinning of Scaffolds with Multidimensional Fiber Architecture for Hierarchical Tissue Engineering. ACS Appl. Mater. Interfaces.

[93] Castilho M., Hochleitner G., Wilson W., van Rietbergen B., Dalton P.D., Groll J., Malda J., Ito K. (2018). Mechanical behavior of a soft hydrogel reinforced with three-dimensional printed microfibre scaffolds. Sci. Rep..

[94] De Ruijter M., Ribeiro A., Dokter I., Castilho M., Malda J. (2019). Simultaneous Micropatterning of Fibrous Meshes and Bioinks for the Fabrication of Living Tissue Constructs. Adv. Healthc. Mater..

[95] Ahmed E.M. (2015). Hydrogel: Preparation, characterization, and applications: A review. J. Adv. Res..

[96] Sánchez-Téllez D.A., Téllez-Jurado L., Rodríguez-Lorenzo L.M. (2017). Hydrogels for Cartilage Regeneration, from Polysaccharides to Hybrids. Polymers.

[97] Zhu J., Marchant R.E. (2011). Design properties of hydrogel tissue-engineering scaffolds. Expert. Rev. Med. Devices.

[98] Fuchs S., Shariati K., Ma M. (2020). Specialty Tough Hydrogels and Their Biomedical Applications. Adv. Healthc. Mater..

[99] Dienes J., Browne S., Farjun B., Amaral Passipieri J., Mintz E.L., Killian G., Healy K.E., Christ G.J. (2021). Semisynthetic Hyaluronic Acid-Based Hydrogel Promotes Recovery of the Injured Tibialis Anterior Skeletal Muscle Form and Function. ACS Biomater. Sci. Eng..

[100] Pepelanova I., Kruppa K., Scheper T., Lavrentieva A. (2018). Gelatin-Methacryloyl (GelMA) Hydrogels with Defined Degree of Functionalization as a Versatile Toolkit for 3D Cell Culture and Extrusion Bioprinting. Bioeng..

[101] Nooeaid P., Salih V., Beier J.P., Boccaccini A.R. (2012). Osteochondral tissue engineering: Scaffolds, stem cells and applications. J. Cell. Mol. Med..

[102] Critchley S., Sheehy E.J., Cunniffe G., Diaz-Payno P., Carroll S.F., Jeon O., Alsberg E., Brama P.A.J., Kelly D.J. (2020). 3D printing of fibre-reinforced cartilaginous templates for the regeneration of osteochondral defects. Acta Biomater..

[103] Doyle S.E., Henry L., McGennisken E., Onofrillo C., Bella C.D., Duchi S., O’Connell C.D., Pirogova E. (2021). Characterization of Polycaprolactone Nanohydroxyapatite Composites with Tunable Degradability Suitable for Indirect Printing. Polymers.

[104] Gong L., Li J., Zhang J., Pan Z., Liu Y., Zhou F., Hong Y., Hu Y., Gu Y., Ouyang H. (2020). An interleukin-4-loaded bi-layer 3D printed scaffold promotes osteochondral regeneration. Acta Biomater..

[105] Jeon J.E., Vaquette C., Theodoropoulos C., Klein T.J., Hutmacher D.W. (2014). Multiphasic construct studied in an ectopic osteochondral defect model. J. R. Soc. Interface.

[106] Kosik-Kozioł A., Heljak M., Święszkowski W. (2020). Mechanical properties of hybrid triphasic scaffolds for osteochondral tissue engineering. Mater. Lett..

[107] Liu X., Liu S., Liu S., Cui W. (2014). Evaluation of oriented electrospun fibers for periosteal flap regeneration in biomimetic triphasic osteochondral implant. J. Biomed. Mater. Res. Part B.

[108] Malikmammadov E., Tanir T.E., Kiziltay A., Hasirci V., Hasirci N. (2018). PCL and PCL-based materials in biomedical applications. J. Biomater. Sci. Polym. Ed..

[109] Mancini I.A.D., Vindas Bolanos R.A., Brommer H., Castilho M., Ribeiro A., van Loon J., Mensinga A., van Rijen M.H.P., Malda J., van Weeren R. (2017). Fixation of Hydrogel Constructs for Cartilage Repair in the Equine Model: A Challenging Issue. Tissue Eng. Part. C. Methods.

[110] Shim J.H., Jang K.M., Hahn S.K., Park J.Y., Jung H., Oh K., Park K.M., Yeom J., Park S.H., Kim S.W. (2016). Three-dimensional bioprinting of multilayered constructs containing human mesenchymal stromal cells for osteochondral tissue regeneration in the rabbit knee joint. Biofabrication.

[111] Di Luca A., Longoni A., Criscenti G., Lorenzo-Moldero I., Klein-Gunnewiek M., Vancso J., van Blitterswijk C., Mota C., Moroni L. (2016). Surface energy and stiffness discrete gradients in additive manufactured scaffolds for osteochondral regeneration. Biofabrication.

[112] Natarajan A.B.M., Sivadas V.P.D., Nair P. (2021). 3D-printed biphasic scaffolds for the simultaneous regeneration of osteochondral tissues. Biomed. Mater..

[113] Thunsiri K., Pitjamit S., Pothacharoen P., Pruksakorn D., Nakkiew W., Wattanutchariya W. (2020). The 3D-Printed Bilayer’s Bioactive-Biomaterials Scaffold for Full-Thickness Articular Cartilage Defects Treatment. Materials.

[114] Reddy M.S.B., Ponnamma D., Choudhary R., Sadasivuni K.K. (2021). A Comparative Review of Natural and Synthetic Biopolymer Composite Scaffolds. Polymers.

[115] Steadman J.R., Rodkey W.G., Singleton S.B., Briggs K.K. (1997). Microfracture technique forfull-thickness chondral defects: Technique and clinical results. Oper. Tech. Orthop..

[116] Wu X., Lian Q., Li D., Jin Z. (2019). Biphasic osteochondral scaffold fabrication using multi-material mask projection stereolithography. Rapid Prototyp. J..

[117] Zhang W., Lian Q., Li D., Wang K., Hao D., Bian W., He J., Jin Z. (2014). Cartilage repair and subchondral bone migration using 3D printing osteochondral composites: A one-year-period study in rabbit trochlea. Biomed. Res. Int..

[118] Zhu S., Chen P., Chen Y., Li M., Chen C., Lu H. (2020). 3D-Printed Extracellular Matrix/Polyethylene Glycol Diacrylate Hydrogel Incorporating the Anti-inflammatory Phytomolecule Honokiol for Regeneration of Osteochondral Defects. Am. J. Sports Med..

[119] Gao F., Xu Z., Liang Q., Liu B., Li H., Wu Y., Zhang Y., Lin Z., Wu M., Ruan C. (2018). Direct 3D Printing of High Strength Biohybrid Gradient Hydrogel Scaffolds for Efficient Repair of Osteochondral Defect. Adv. Funct. Mater..

[120] Liu J., Li L., Suo H., Yan M., Yin J., Fu J. (2019). 3D printing of biomimetic multi-layered GelMA/nHA scaffold for osteochondral defect repair. Mater. Des..

[121] Liu X., Wei Y., Xuan C., Liu L., Lai C., Chai M., Zhang Z., Wang L., Shi X. (2020). A Biomimetic Biphasic Osteochondral Scaffold with Layer-Specific Release of Stem Cell Differentiation Inducers for the Reconstruction of Osteochondral Defects. Adv. Healthc. Mater..

[122] Moses J.C., Saha T., Mandal B.B. (2020). Chondroprotective and osteogenic effects of silk-based bioinks in developing 3D bioprinted osteochondral interface. Bioprinting.

[123] Zhang H., Huang H., Hao G., Zhang Y., Ding H., Fan Z., Sun L. (2021). 3D Printing Hydrogel Scaffolds with Nanohydroxyapatite Gradient to Effectively Repair Osteochondral Defects in Rats. Adv. Funct. Mater..

[124] Bellucci D., Sola A., Cannillo V. (2016). Hydroxyapatite and tricalcium phosphate composites with bioactive glass as second phase: State of the art and current applications. J. Biomed. Mater. Res. Part A.

[125] Szychlinska M.A., Bucchieri F., Fucarino A., Ronca A., D’Amora U. (2022). Three-Dimensional Bioprinting for Cartilage Tissue Engineering: Insights into Naturally-Derived Bioinks from Land and Marine Sources. J. Funct. Biomater..

[126] Zhang Y., Zhou D., Chen J., Zhang X., Li X., Zhao W., Xu T. (2019). Biomaterials Based on Marine Resources for 3D Bioprinting Applications. Mar. Drugs.

[127] Eschweiler J., Horn N., Rath B., Betsch M., Baroncini A., Tingart M., Migliorini F. (2021). The Biomechanics of Cartilage—An Overview. Life.

[128] Goh K.L., Holmes D.F. (2017). Collagenous Extracellular Matrix Biomaterials for Tissue Engineering: Lessons from the Common Sea Urchin Tissue. Int. J. Mol. Sci..

[129] Galli S., Stocchero M., Andersson M., Karlsson J., He W., Lilin T., Wennerberg A., Jimbo R. (2017). The effect of magnesium on early osseointegration in osteoporotic bone: A histological and gene expression investigation. Osteoporos. Int..

[130] Li M., He P., Wu Y., Zhang Y., Xia H., Zheng Y., Han Y. (2016). Stimulatory effects of the degradation products from Mg-Ca-Sr alloy on the osteogenesis through regulating ERK signaling pathway. Sci. Rep..

[131] Kao F.-C., Chiu P.-Y., Tsai T.-T., Lin Z.-H. (2019). The application of nanogenerators and piezoelectricity in osteogenesis. Sci. Technol. Adv. Mater..

[132] deVente J.E., Lester G.E., Trotter J.A., Dahners L.E. (1997). Isolation of intact collagen fibrils from healing ligament. J. Electron. Microsc..

[133] Wilkie I.C., Sugni M., Gupta H.S., Carnevali M.D.C., Elphick M.R. (2021). Chapter 1 The Mutable Collagenous Tissue of Echinoderms: From Biology to Biomedical Applications. Soft Matter for Biomedical Applications.

[134] Gallo N., Natali M.L., Sannino A., Salvatore L. (2020). An Overview of the Use of Equine Collagen as Emerging Material for Biomedical Applications. J. Funct. Biomater..

[135] Angele P., Abke J., Kujat R., Faltermeier H., Schumann D., Nerlich M., Kinner B., Englert C., Ruszczak Z., Mehrl R. (2004). Influence of different collagen species on physico-chemical properties of crosslinked collagen matrices. Biomaterials.

[136] Terzi A., Storelli E., Bettini S., Sibillano T., Altamura D., Salvatore L., Madaghiele M., Romano A., Siliqi D., Ladisa M. (2018). Effects of processing on structural, mechanical and biological properties of collagen-based substrates for regenerative medicine. Sci. Rep..

[137] Miller T., Goude M.C., McDevitt T.C., Temenoff J.S. (2014). Molecular engineering of glycosaminoglycan chemistry for biomolecule delivery. Acta Biomater..

[138] Dinoro J., Maher M., Talebian S., Jafarkhani M., Mehrali M., Orive G., Foroughi J., Lord M.S., Dolatshahi-Pirouz A. (2019). Sulfated polysaccharide-based scaffolds for orthopaedic tissue engineering. Biomaterials.

[139] Phull A.R., Kim S.J. (2017). Fucoidan from Undaria pinnatifida regulates type II collagen and COX-2 expression via MAPK and PI3K pathways in rabbit articular chondrocytes. Biologia.

[140] Liacini A., Sylvester J., Li W.Q., Zafarullah M. (2005). Mithramycin downregulates proinflammatory cytokine-induced matrix metalloproteinase gene expression in articular chondrocytes. Arthritis Res. Ther..

